# Cocaine-induced destruction of the palate: a diagnostic and management challenge

**DOI:** 10.1038/s41415-024-7834-5

**Published:** 2024-09-27

**Authors:** Brian Maloney, Kate Hinchion, Niall Conlon, Osama Omer, Dermot Pierse

**Affiliations:** 130006820529602356863 Dublin Dental University Hospital, Trinity College Dublin, Ireland; 480510196140993219476 St. James´s Hospital, Dublin, Ireland

## Abstract

Cocaine usage is increasing at a rate faster than population growth worldwide. The habitual and chronic insufflation of pulverised cocaine is associated with the progressive destruction of the osseocartilaginous structures of the midface, termed cocaine-induced midline destructive lesions (CIMDLs). These entities present a challenging diagnostic picture, mimicking other infectious, malignant and inflammatory conditions associated with midface destruction. CIMDLs can present along a wide spectrum of disease, with minimal palatal perforation to extensive sinonasal destruction. With the increasing usage of cocaine, there is likely to be a concurrent increase in patients presenting to emergency departments with these destructive entities. Therefore, there is a need to create awareness of this uncommon entity and to document a systematic approach that must be adopted to reach a definitive diagnosis which will subsequently inform management. We report three clinical cases of CIMDLs at varying stages of the disease process which presented to the Dublin Dental University Hospital between January 2023 and June 2024 and document their multidisciplinary management from initial presentation to eventual treatment.

## Key points

- Aims to create an awareness among dental practitioners of the clinical features of this destructive process as with increasing cocaine use worldwide, patients presenting with these conditions are likely to increase.
- Highlights the complex diagnostic process for CIMDL, ruling out malignancy and vasculitis.
- Will create an awareness amongst the public of an increasingly common form of midline destructive lesion, while also highlighting the other aetiologies associated with this clinical presentation.

## Introduction

Cocaine is a vasoactive tropane alkaloid derived from the Erythroxylon plant which was introduced into medical practice in the nineteenth century for its combined anaesthetic and vasoconstrictive properties.^1^ The potent psychostimulant effects of the drug, which arise from dopamine reuptake inhibition at the level of the synaptic cleft, have resulted in cocaine becoming the most widely used illicit drug worldwide.^2^ The highly addictive nature of the drug, coupled with its psychosocial and organic side effects, presents an emerging global public health problem.

Cocaine can be administered orally, intravenously, intranasally or be inhaled, and elicits idiosyncratic, deleterious, multisystem manifestations affecting the cardiovascular, neurological, ophthalmologic and respiratory systems. Cocaine use can also have local effects which are less well-documented and understood.

The impact of cocaine on the nasal cavity has been highlighted as early as 1912.^3^ Locally, the habitual nasal insufflation of pulverised cocaine is associated with inflammation, ischaemia and eventual necrosis of the lining of the nasal cavity and paranasal air sinuses. Repeated and chronic use results in the progressive destruction of the septal cartilage and the formation of palatal perforation. In severe cases, there is destruction of the osseocartilaginous structures that form the palate, paranasal air sinuses and nose, alongside a loss of midline facial support structures, culminating in deformities of the facial mid-third and nose with concurrent infections.

These so-called cocaine-induced midline destructive lesions (CIMDLs) may display a clinical picture mimicking other causes of necrotising midfacial lesions, with positive antinuclear cytoplasmic antibodies, and similar radiographic and histological findings as tumours, infections and immunological disorders. Additionally, no single diagnostic test can attribute CIMDL to cocaine usage. Consequently, the diagnosis of CIMDL must consider other differential diagnoses and follow a systematic diagnostic approach to reach a diagnosis which will govern further management.

We report four clinical cases of CIMDLs at varying stages of the disease process. They presented to the Dublin Dental University Hospital (DDUH) between January 2023 and May 2024 and we document their multidisciplinary management from initial presentation to eventual treatment.

## Clinical cases

### Case 1

This patient in their thirties was referred to the DDUH in July 2023. The patient reported a history of chronic cocaine abuse for several years. The patient had developed a palatal fenestration which was impacting speech. Medically, the patient was fit and well.

Previous investigations for this patient included multiple soft tissue biopsies of the midline defect under general anaesthesia, with samples taken from the site of the nasal perforation margin, sinusoidal cavities and hard palate margin, which returned a non-specific result but were clear of vasculitis and malignancy. Previous management included a complement of blood tests to assess for vasculitis (full blood count [FBC], c-reactive protein [CRP], antineutrophil cytoplasmic antibodies [ANCA], myeloperoxidase [MPO]) which were not significant. The patient's referral to the DDUH was for the interim prosthetic rehabilitation of their palatal fenestration.

**Fig. 1:**
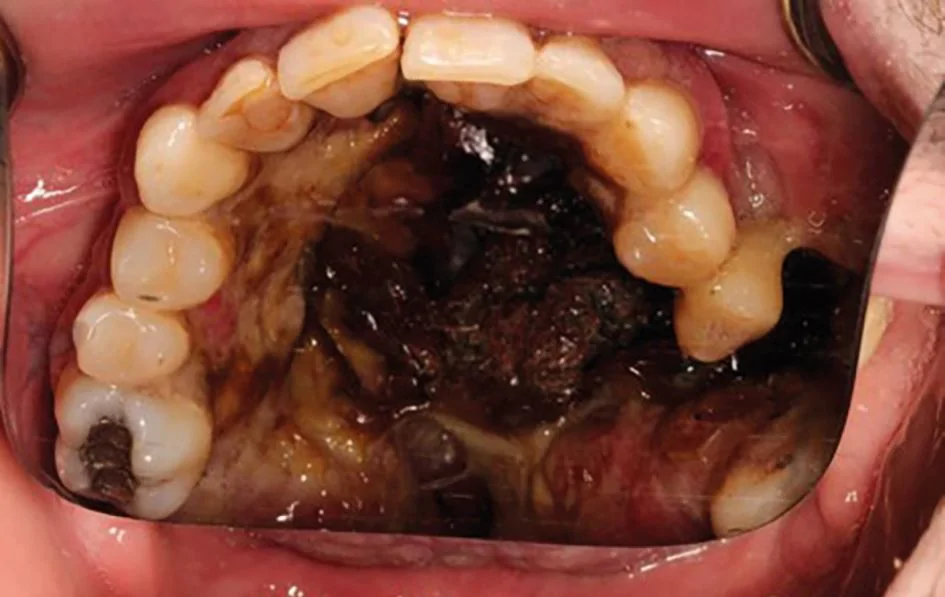
CIMDL with extensive oral and nasopharyngeal destruction

**Fig. 2:**
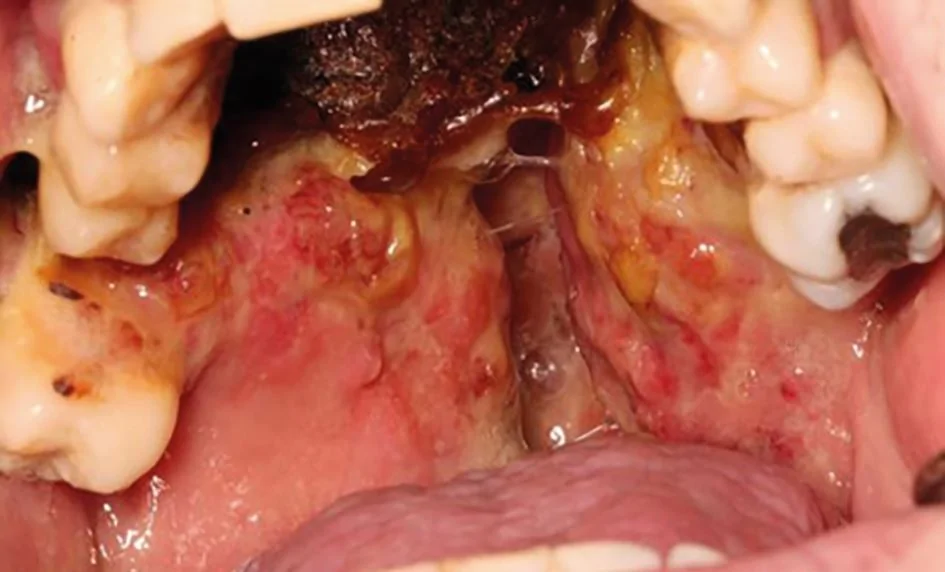
CIMDL with extensive oral and nasopharyngeal destruction

**Fig. 3:**
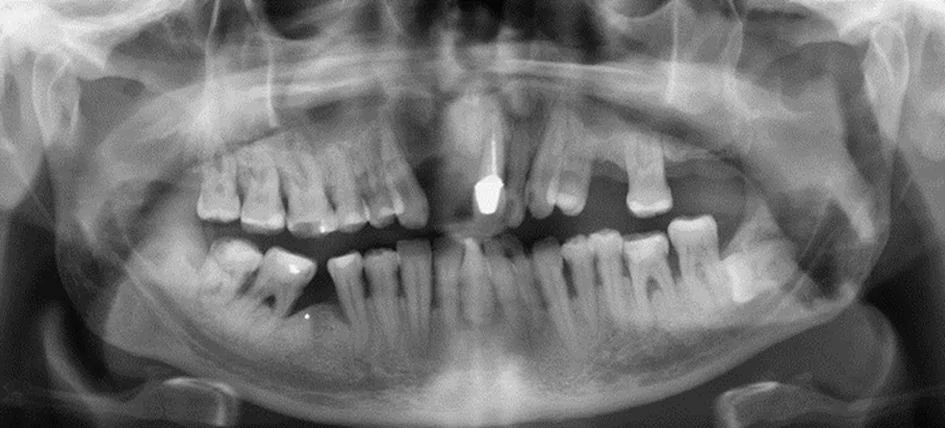
Orthopantomogram of the patient’s dentition

On examination, the patient had a visible lack of nasal columella but no saddle nose deformity (Fig. 1). Intra-orally, there was a fenestration of the left hard palate with direct communication with the nasal cavity. There was an absence of erythema or crusting surrounding the defect (Fig. 2, Fig. 3).

**Fig. 4:**
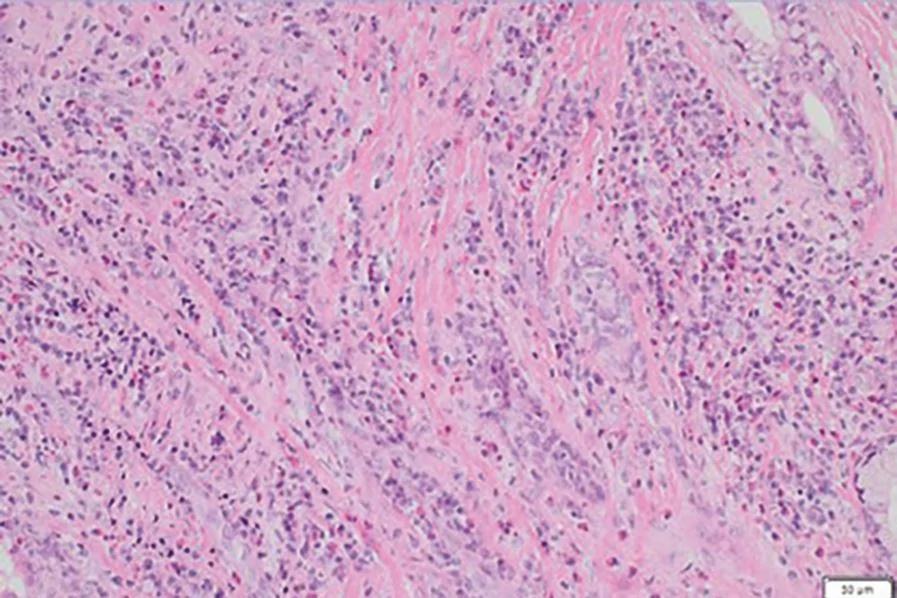
Histopathology of biopsy specimen from palatal tissue (report: non-specific inflammatory process, characterised by dense interstitial inflammatory infiltrate, no evidence of granulomas, and negative for syphilis, fungal infection, or Epstein-Barr virus. There was an increase in IgG4+ plasma cells, raising the suspicion of IgG4-related disease. The sample was negative for vasculitis, lymphoma, and malignancy)

Treatment involved counselling on cessation of cocaine use and the construction of a palatal obturator. The patient reported continued cessation of cocaine use and reported significantly improved quality of life. However, there was no follow-up from substance abuse services to confirm this. On review, the patient reported continued use of cocaine and that the palatal fistula had increased in size (Fig. 4). The patient recently tested positive for autoantibodies against proteinase-3 (PR3) and myeloperoxidase (MPO). The patient is under follow-up with immunology but no further treatment is planned other than drug cessation.

### Case 2

Case 2 involves a patient in their late twenties who was seen in the emergency department of the DDUH in January 2023. The patient presented with discomfort, pain and difficulty speaking due to a cocaine-induced midline intranasal and oropharyngeal destructive process. Previous biopsies of the site had ruled out malignancy. Surgical repair of the defect was deemed not feasible. Medically, the patient had eczema, asthma and hayfever. The patient was a current smoker of 20 cigarettes per day and consumed 30 units of alcohol per week. A seven-year history of cocaine abuse was revealed.

**Fig. 5:**
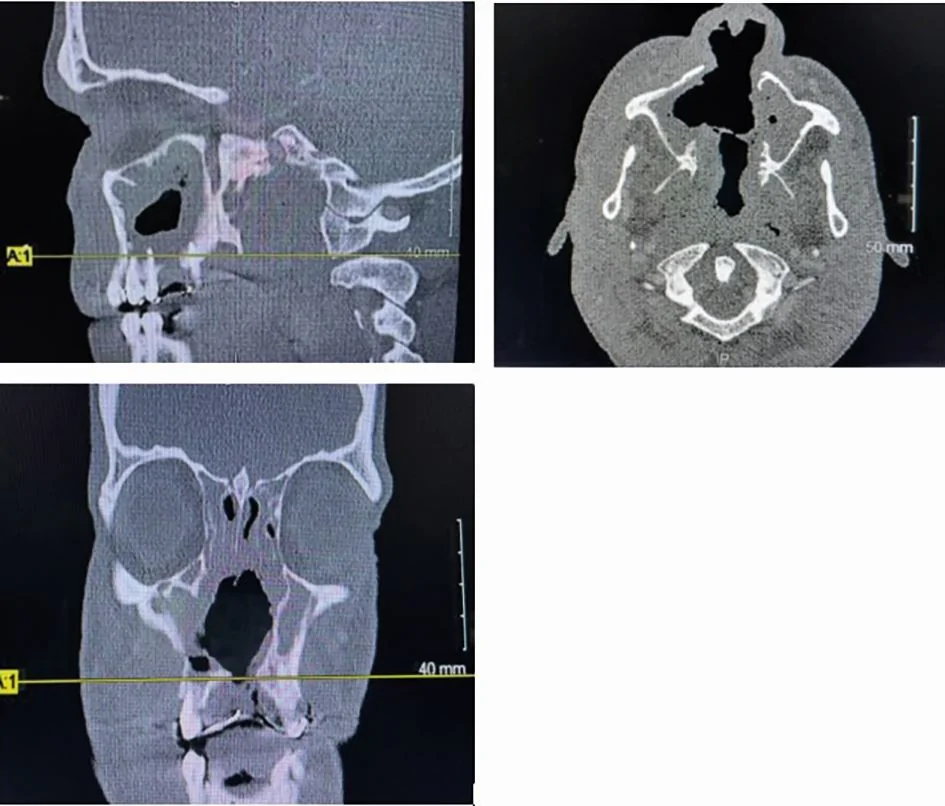
Head and neck CT for Case 1, highlighting the extent of the CIMDL

**Fig. 6:**
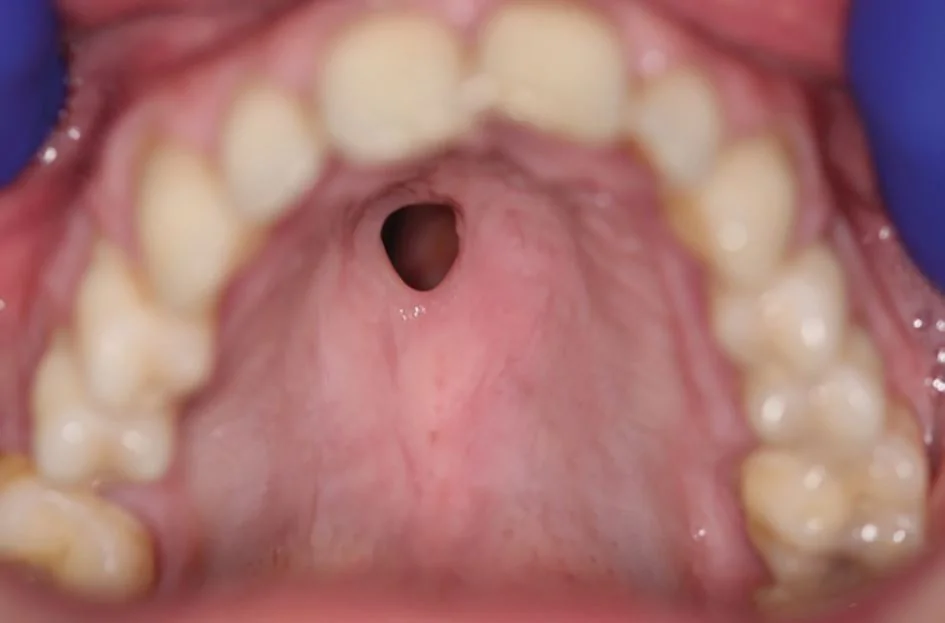
Palatal defect associated with cocaine abuse

**Fig. 7:**
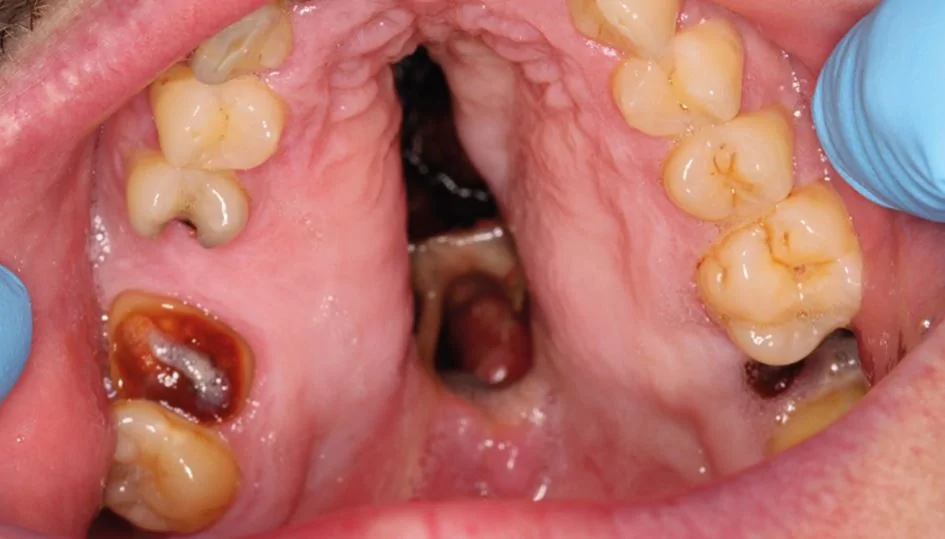
Palatal fenestration secondary to cocaine use

On examination, there was a loss of the nasal columella and saddle nose deformity. Intra-orally, there was complete erosion of the hard and soft palate and destruction of the nasal septum, with superimposed overgrowth of tissue and suppuration extending from the anterior hard palate posteriorly toward the oropharynx. The defect was surrounded with florid angiomatous tissue and bordered with crusted necrotic tissue (Fig. 5, Fig. 6). All remaining maxillary teeth were mobile (Fig. 7).

Management for this patient involved an urgent biopsy of the palatal tissue. In addition, there was a concerning ulcerated area of the buccal mucosa which was also biopsied. A panel of blood tests was carried out to assess for vasculitis and agranulocytosis associated with levamisole adulterated cocaine (FBC; haematinic; CRP; liver and renal profile; connective tissue disease screen; c-ANCA [cytoplasmic staining antineutrophil cytoplasmic antibodies]; p-ANCA [perinuclear/nuclear staining antineutrophil cytoplasmic antibodies]; PR3 [proteinase 3]; glucose; albumin; rheumatoid factor; immunoglobulin levels; glomerular basement membrane; thyroid peroxidase).

**Fig. 8:**
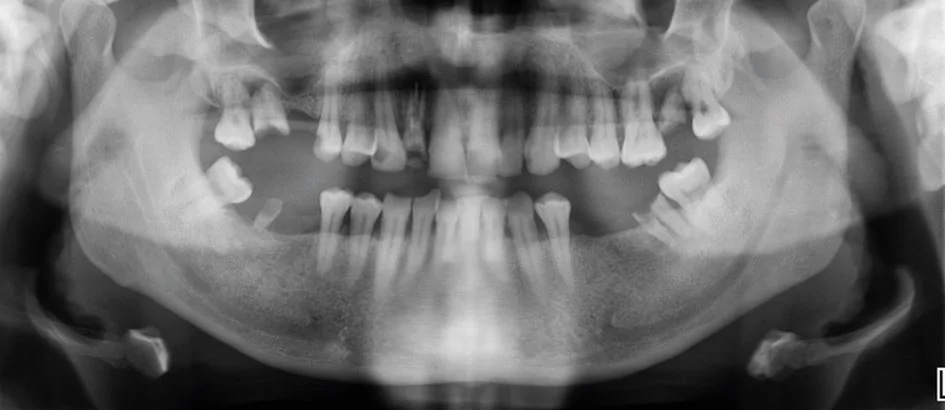
Radiograph of dentition of Case 3

Significant findings included an elevated erythrocyte sedimentation rate (ESR) of 30 (0-15 mm/hr), CRP of 33.10 (0-5 mg/L), elevated immunoglobulin A (IgA) of 3.27 (0.62-2.90 g/L), and immunoglobulin G (IgG) of 16.48 (6.26-14.96 g/L). Anti-PR3 was positive at 71 (0-1.9 IU/ml) and c-ANCA was weakly positive. Myeloperoxidase was negative. The biopsy of the palatal tissue demonstrated a non-specific inflammatory process, which was negative for granuloma (excluding tuberculous and sarcoidosis), syphilis, fungal infection, vasculitis, lymphoma and malignancy (Fig. 8).

The right buccal mucosa sample revealed a non-specific intraepithelial eosinophilic inflammation. The differential diagnosis includes granulomatosis with polyangiitis (GPA), eosinophilic GPA, other autoimmune and inflammatory disorders, and sequelae of intranasal drugs and toxins.

Management for this patient included stabilising their dental condition followed by medical and surgical interventions. An upper denture was made as an interim measure to act as an obturator for the defect. The patient also required multiple extractions of teeth involved in the CIMDL defect.

The presence of ANCA raised suspicion of systemic vasculitis. The patient was referred to the immunology department in St James's Hospital, Dublin, for concurrent care. Subsequent investigations included a computed tomography (CT) head extension of the CIMDL into the maxillary sinuses but no involvement of the orbital region (Fig. 5). Repeat blood investigations showed consistently high CRP, IgA, IgG and a positive c-ANCA with elevated levels of anti-PR3 antibodies. Attempts were made to link the patient with an addiction services program, but they declined help on multiple occasions. Repeated urine toxicology demonstrated continued use of cocaine despite the patient reporting abstinence.

The patient's failure to comply with drug cessation and the extent of destruction deemed them unsuitable for surgery to repair the defect. Continued drug use presented a challenge for immune suppression. The patient is still under review and follow-up in the Dublin Dental Hospital and the immunology department of St James's Hospital, Dublin.

### Case 3

This patient in their thirties presented to the emergency department of the DDUH in September 2023 with a complaint of palatal perforation associated with cocaine usage. The patient's primary complaint was regurgitation of fluids into the nose from the mouth. A recent biopsy of the anterior ethmoid region excluded malignancy and vasculitis. The patient's medical history was significant for asthma. The patient smoked 20 cigarettes per day for 18 years and consumed 50 units of alcohol per week. A cocaine habit of more than ten years was also revealed.

On examination, there was marked flattening of the bridge of the nose. There was no evidence of cervical lymphadenopathy. Intra-orally, there was a palatal fenestration communicating with the nasal cavity (Fig. 6).

An interim splint vacuum-form retainer was made to close the defect initially. In the long-term, the patient will require an obturator appliance once poor prognosis teeth in the maxilla are extracted.

### Case 4

A patient was referred to the DDUH in May 2024 regarding prosthetic treatment for a palatal fenestration secondary to drug use. The midline defect was first noticed severed a month previously but had increased in size and was impacting speech. Investigations, including a biopsy of the site, which excluded malignancy and vasculitis. The patient's two-year history of cocaine use resulted in the diagnosis of a CIMDL. Medically, the patient was fit and well. The patient was a smoker of 25 cigarettes per day and consumed 40 units of alcohol per week.

Examination revealed a saddle nose deformity and absence of nasal septum. Intra-orally, there was a midline palatal fenestration with communication into the nasal cavity (Fig. 7, Fig. 8).

Treatment for this patient was limited to fabrication of an obturator. Pending continued drug cessation, the patient may be suitable for surgical reconstruction of the defect. The patient is currently being reviewed by otorhinolaryngology for ongoing care and progression of the lesion.

## Discussion

The global prevalence of cocaine abuse has been steadily increasing since 1990. The European Drug Report 2023 reports that cocaine is the second most commonly used drug in Europe, with 2.3 million 15-34-year-olds (2.3%) abusing the drug,^4^ and is responsible for one in five overdose deaths. Ireland is the joint-fourth highest consumer of cocaine globally, with 2.4% of the population using the drug. The increasingly widespread use of cocaine in Ireland and globally will undoubtedly result in a rise in cocaine-associated pathology.

The first link between cocaine-inducing autoimmunity was highlighted by two reports of destructive midface sinonasal lesions in patients with ANCA-positive serum.^5^^,^^6^ The true incidence of oral cocaine-related complications worldwide is unknown. The incidence of nasal septum perforation - the most common adverse effect in cocaine users - is reported to be 4.8%.^7^ However, this is likely to be an underestimation.

CIMDLs have a complex and multifactorial pathogenesis.^8^ The proposed process has been described by Smith *et al*. (2002). In the incipient stages of the disease process, there is localised damage to the tissues, as a result of exposure to cocaine crystals.^9^ The addition of adulterants causes chemical irritation of the mucosal lining, leading to inflammation and ulceration.^10^ The vasoconstrictor effect of the drug results in ischaemia, which, if prolonged, induces cellular apoptosis, which in turn may stimulate an immunologic reaction.^11^ This ultimately culminates in exposing tissues to inflammation and irritation. When CIMDLs become more extensive, the open lesions are highly susceptible to superinfection.^12^

According to Smith *et al*., the diagnosis of CIMDLs is based on the presence of at least two of the following features: nasal septal perforation, lateral nasal wall destruction and hard palate involvement.^11^ Patients initially report long-standing persistent local symptoms associated with this condition. Early lesions result in nasal obstruction, facial pain and epistaxis in the absence of systemic manifestations.^7^ Clinical features of established, long-standing CIMDLs are extensive and variable, as seen in the presented cases. As these lesions progress rapidly, they become cavitated and are bordered with ulcerated and crusted mucosa with necrotic debris. There is common destruction of the osseocartilaginous structures of the nose maxilla and palate, including partial or total nasal septal perforation (seen in all cases) and destruction of the turbinates. Advanced disease involves nasal conchae allowing infection to spread to the sphenoid and orbit.^13^ The destruction results in extensive deformities of the facial mid-third and nose, as seen in each of the three cases above.

The clinical course of CIMDLs, if unabated, will continue to extend centrifugally to destroy structures beyond the nasal and oral cavities. Several reports have documented the spread to the orbit, with effects ranging from dehiscence of the orbit floor to optic neuropathy and pseudotumour.^14^^,^^15^^,^^16^ Involvement of the base of the skull resulting in encephalocele has been reported in three cases.^15^^,^^17^^,^^18^ One case progressed to pneumocephalus^19^ and another to pituitary gland damage and panhypopituitarism.^20^

Midline destructive sinonasal lesions have numerous aetiologies. One of the diagnostic challenges of CIMDLs is their misleading clinical presentation. The signs/symptoms of CIMDLs are often not determinant and mimic the appearance of various aetiologists of midfacial sinonasal destruction, including aggressive inflammatory, infective and neoplastic diseases. Patients are reluctant to admit a cocaine addiction which further complicates diagnosis and requires a comprehensive diagnosis protocol to inform treatment. Certain pathologies may be easily excluded through history, blood tests and biopsies (Box 1). One of the most important differential diagnoses to consider is GPA. GPA shares several similarities with CIMDLs, from its clinical presentations to histological and serological findings. GPA is characterised by inflammation of the upper and lower airways with small/medium blood vessel vasculitis with glomerulonephritis. It is seen most commonly in middle age and often presents with nasal signs/symptoms. While CIMDL has local effects, GPA can also be localised to the upper airway only, without systemic involvement. There are also some different features seen in histology and serology which help to exclude this aggressive pathology. However, excluding GPA can be difficult and a high index of suspicion is important before reaching a definitive diagnosis.

Levamisole is a veterinary anthelminthic and a synthetic derivative of imidazothiazole, and is the primary adulterant in cocaine.^21^ Since 2005, this form of drug-induced vasculitis has been recognised. This entity has significant overlap in clinical, serological, radiographic and histological features with CIMDLs, as well as with GPA, further complicating diagnosis, characterised by p-ANCA and antinuclear antibodies, alongside agranulocytosis and extensive necrotic lesions and retiform purpura, often affecting the earlobes.^2^ Levamisole-induced vasculitis may also result in lung haemorrhage and glomerulonephritis. A further challenge with the diagnosis of this entity is the short half-life of the drug, as well as the failure to detect it on standard toxicology.^22^

Destructive sinonasal processes suggest the classic differential diagnostic spectrum, ranging from inflammatory processes to malignant neoplasia. Diagnosis of CIMDLs, however, is challenging and requires a structured approach (Box 2). The first stage in managing this cohort of patients involves a comprehensive history and systematic physical examination.

A history of the pathology should elucidate its onset, evolution and patient-reported symptoms. Systemic symptoms should also be queried, including respiratory problems, malaise, weight loss, skin rash myalgia and peripheral neuropathy, which would raise suspicion of GPA. A complete up-to-date medical history is also necessary as part of the workup. A thorough history of recreational drug abuse is important to elicit in cases of suspected CIMDLs, including frequency and quantity of usage, and previous attempts to quit. Obtaining an accurate history of drug use, however, is often difficult to obtain.

The physical examination includes extra- and intra-oral assessments. Extra-oral features of note in cases of CIMDLs include saddle nose deformity and loss of septum. An intra-oral exam should examine the hard and soft tissues, as well as the extent of the lesion.

Following thorough clinical assessment, CIMDLs will be only one differential for midfacial destructive pathologies (Box 1). The information collected from the patient history and assessment will allow the clinician to generate a list of appropriate differentials. Further investigations will be necessary to reach a definitive diagnosis which will guide treatment and will vary depending on the suspected aetiology of the condition.

Additional investigations for destructive midface defects include radiological imaging and laboratory tests, including bloods and histological assessment of sample tissues. Imaging of CIMDLs is often appropriate in cases of extensive destruction to determine the degree of spread and tissues affected. Modalities include CT and MRI (magnetic resonance imaging), the former being more appropriate for assessment of bony erosion.^23^ Chest x-ray may be necessary to exclude tuberculosis or GPA, which was ordered in Case 1. Laboratory investigations useful for the diagnosis of CIMDLs include a FBC, CRP and various markers of systemic inflammation of blood vessels, including ANA (antinuclear antibody) for lupus. Serology may be justified if a fungal or bacterial cause is suspected.

An important test specific to autoimmune-medicated CIMDLs includes the identification of ANCA, which is useful to differentiate sinonasal defects secondary to cocaine and vasculitis. There is a growing body of evidence implicating cocaine as a trigger for anti-neutrophil cytoplasmic antibody-associated vasculitis, which can mimic idiopathic GPA, as in Case 1.^24^ ANCA are a type of autoreactive antibodies which attack specific antigens located in the cytoplasm of granulocytes. Serology can identify two distinct patterns: c-ANCA and p-ANCA. C-ANCA is directed against PR3 while p-ANCA is directed against MPO. The incidence of p- and c-ANCA are 60% and 40%, respectively, in patients with CIMDLs. In addition, ANCA for neutrophil elastase has been identified as a valuable diagnostic marker for CIMDLs. This marker demonstrates high sensitivity for CIMDLs, seen in 70-85% of cases. This marker is also not seen in GPA, thus helping to differentiate between the two pathologies.^25^

While tissue biopsy is advised to support the clinical diagnosis of CIMDLs, the histopathologic differentiation of CIMDLs from other pathologies, specifically GPA, is challenging and should not be relied on solely. These conditions tend to share similar, non-specific histological features. GPA has certain pathognomonic features, which are necessary for diagnosis; however, these features are only seen in 50% of cases, and definitive diagnosis cannot be made based on histology alone.

The treatment of CIMDLs is challenging and based on weak evidence, given the scarcity of high-quality reports with adequate participants and long follow-up in the literature. Initial therapy should be focused on the resolution of the acute lesions, including curettage and debridement, antibiotics, and nasal lavage to eliminate foci of infection. Once the acute condition is controlled and the patient has demonstrated consistent abstinence, there are options for prosthetic or surgical defect repair. However, the surgical reconstruction of the hard and soft tissues partially or fully destroyed by cocaine is a complex task.^26^ The availability of viable tissue in the region of the CIMDL is poor. This is compounded by the poor vascularity of local tissue resulting in rendering graft uptake less favourable.^26^ Treatment is further complicated by poor compliance of patients^15^ and failure to abstain from drug usage. Surgery should not be considered in the presence of an ongoing cocaine habit, as every and all techniques will ultimately fail. Potent immunosuppression is necessary for the treatment of true idiopathic vasculitis such as GPA. However, in the management of CIMDL, there is contrasting evidence for the benefit of this form of therapy, as drug cessation in early cases may be sufficient for clinical resolution.^27^

Patients with CIMDLs often demonstrate poor compliance with cocaine cessation.^15^ Failure to demonstrate sustained abstinence renders any surgical repair of these defects unfeasible. Drug counselling services are therefore an important component of the multidisciplinary management of patients with CIMDLs. Patients should be followed-up regularly for regular toxicology to ensure abstinence is sustained.

Box 1 Differential diagnosis for midline destructive lesions. Adapted with permission from N. P. Parker *et al.*, ‘The dilemma of midline destructive lesions: a case series and diagnostic review', *American Journal of Otolaryngology*, 2010, vol 31, pp 104-109^28^ Infectious

- Bacterial: syphilis, tuberculosis, actinomycosis, leprosy, rhinoscleroma
- Fungal: histoplasmosis, mucormycosis, blastomycosis, aspergillosis

Neoplastic

- Adenoid cystic carcinoma
- Squamous cell carcinoma
- Extranodal natural killer/T-cell lymphoma
- Rhabdomyosarcoma

Drug-induced

- Cocaine-induced
- Levamisole-induced

Autoimmune

- Granulomatosis with Polyangitis (GPA)
- Sarcoidosis
- Systemic lupus erythematosus

Box 2 Diagnostic protocol for CIMDLs Thorough history of complaint

- Onset
- Duration
- Associated symptoms: local (epistaxis, nasal congestion/obstruction, crusting of the nose, facial pain) and/or systemic (weight loss, fever, malaise)

Medical history

Social history, including a history of substance abuse

- Onset and duration
- Previous quit attempts
- Liaison with addiction services

Examination

- Thorough extra- and intra-oral examination to assess for
  - Saddle nose deformity
  - Perforated septum
  - Palatal fenestration; site and extent

Blood panel to rule out other causes of MDL including:

- FBC: leukocytosis (chronic infection, levamisole-induced MDL)
- ESR/CRP: general markers of inflammation
- c- and p-ANCA, MPO, PR3 autoantibodies (GPA, CIMDL)
- SACE (sarcoidosis) (serum angiotensin-converting enzyme)
- Anti-ANA, anti-Ro/La, anti-Smith (lupus)

Biopsy of tissue from MDL for:

- Histopathology
- Immunofluorescence

Imaging

- Dental imaging: orthopantomogram
- Medical imaging: CT, MRI, chest x-ray (to rule out sarcoid, tuberculosis)

Nasal cultures

- To assess for bacterial (actinomyces, bacilli) and fungal growth

Other investigations

- Treponemal and non-treponemal serology; syphilis
- Ziehl-Neelsen; tuberculosis

## Conclusion

The global rise in cocaine abuse will ultimately result in a greater number of patients presenting to emergency departments with cocaine-induced midfacial lesions. Diagnosis is challenging since various conditions (mainly vasculitis pathologies) may mimic this acquired condition. We present three cases of CIMDLs with varying stages of disease progression.

It is important to consider CIMDL as a differential for midfacial destructive processes and to follow a diagnostic protocol, including a thorough medical and drug history, clinical examination, serology, and radiographic and histological assessment, to rule out other conditions.

The management of these cases is currently based on weak evidence from case reports and series' with small populations and inadequate follow-up. Future well-designed prospective trials with sufficient participants are essential before evidence-based recommendations can be made in the management of these destructive lesions.
