# Correction to: Cocaine-induced destruction of the palate: a diagnostic and management challenge

**DOI:** 10.1038/s41415-026-9945-7

**Published:** 2026-07-24

**Authors:** Brian Maloney, Kate Hinchion, Niall Conlon, Osama Omer, Dermot Pierse

**Affiliations:** 055392859380300146513 Dublin Dental University Hospital, Dublin, Ireland; 645700628733200517648 St. James´s Hospital, Dublin, Ireland; 657533477763787234089 Dublin Dental University Hospital, Trinity College Dublin, Ireland

Clinical. *Br Dent J* 2024; **240:** 6

The article has been corrected to remove a case report as a patient withdrew their consent for publication.

